# Quantitative texture analysis based on dynamic contrast enhanced MRI for differential diagnosis between primary thymic lymphoma from thymic carcinoma

**DOI:** 10.1038/s41598-022-16393-y

**Published:** 2022-07-24

**Authors:** Jia-jia Zhu, Jie Shen, Wei Zhang, Fen Wang, Mei Yuan, Hai Xu, Tong-fu Yu

**Affiliations:** Department of Radiology, Jiangsu Province People’s Hospital, Nanjing Medical University First Affiliated Hospital, No. 300 Guangzhou Road, Nanjing, 210029 Jiangsu People’s Republic of China

**Keywords:** Cancer, Medical research

## Abstract

To evaluate the value of texture analysis based on dynamic contrast enhanced MRI (DCE-MRI) in the differential diagnosis of thymic carcinoma and thymic lymphoma. Sixty-nine patients with pathologically confirmed (thymic carcinoma, n = 32; thymic lymphoma, n = 37) were enrolled in this retrospective study. K^trans^, K_ep_ and V_e_ maps were automatically generated, and texture features were extracted, including mean, median, 5th/95th percentile, skewness, kurtosis, diff-variance, diff-entropy, contrast and entropy. The differences in parameters between the two groups were compared and the diagnostic efficacy was calculated. The K^trans^-related significant features yielded an area under the curve (AUC) of 0.769 (sensitivity 90.6%, specificity 51.4%) for the differentiation between thymic carcinoma and thymic lymphoma. The K_ep_-related significant features yielded an AUC of 0.780 (sensitivity 87.5%, specificity 62.2%). The V_e_-related significant features yielded an AUC of 0.807 (sensitivity 75.0%, specificity 78.4%). The combination of DCE-MRI textural features yielded an AUC of 0.962 (sensitivity 93.8%, specificity 89.2%). Five parameters were screened out, including age, K^trans^-entropy, K_ep_-entropy, V_e_-entropy, and V_e_-P95. The combination of these five parameters yielded the best discrimination efficiency (AUC of 0.943, 93.7% sensitivity, 81.1% specificity). Texture analysis of DCE-MRI may be helpful to distinguish thymic carcinoma from thymic lymphoma.

## Introduction

Thymic carcinoma and thymic lymphoma are the two most common malignant lesions in the anterior mediastinal region^1^. The clinical manifestations of solid anterior mediastinal masses are usually non-specific, and there is considerable overlap between the imaging manifestation of thymic lymphoma and thymic carcinoma^2^. Differentiation using conventional CT or MRI is challenging and both are subjective with low repeatability. The treatment of thymic cancer is mostly surgical resection, combined with comprehensive treatment of radiotherapy and chemotherapy, while chemotherapy is the first choice for thymic lymphoma^3,4^. Accurate identification of thymic carcinoma and thymic lymphoma is fundamental for the pre-treatment diagnosis. Histopathology is the gold standard for the diagnosis of mediastinal tumours; however, the sampling of needle biopsy is relatively limited, which cannot reflect the whole lesion^5^. Imaging examination can evaluate the lesion as a whole. Therefore, an accurate and reliable imaging method for the evaluation of solid anterior mediastinal tumors is urgently needed in clinical practice.

Several studies have described specific imaging approaches that may be of value in the diagnosis of thymic tumors. CT has previously been a common method for the identification of thymic tumors, and the value of CT perfusion imaging and energy spectrum imaging in the differential diagnosis of thymic carcinoma and lymphoma has been demonstrated^2,6,7^. Compared with CT, MRI is playing an increasingly important role in disease diagnosis, especially functional MRI, due to its accurate assessment of tumor location, expansion method and anatomical relationship with adjacent structures of the disease^1^. Many studies have been conducted on the differentiation and staging of mediastinal masses by diffusion weighted imaging (DWI)^8–10^. Previous studies have shown that the apparent diffusion coefficient (ADC) value of high-risk thymomas and thymic carcinoma was lower than that of low-risk thymomas, and the ADC value of advanced (stage III and IV) thymomas was lower than that of early (stage I and II) thymomas^9^. Zhang et al.^10^ showed that ADC histogram had high diagnostic efficacy in distinguishing thymic epithelial tumor from thymic lymphoma, and confirmed that thymic lymphoma had lower ADC value. However, some scholars have found no significant difference in ADC values between the two groups of anterior mediastinal thymic epithelial tumor and thymic lymphoma^11,12^. The differential ability of DWI for thymic carcinoma and thymic lymphoma remains controversial. And the routine DWI sequence is susceptible to gas and movement, which largely limits its application in mediastinal lesions^13^. In recent years, researches on deep learning and radiomics have become a hot spot in imaging research^14,15^. However, due to the relatively low incidence of mediastinal tumors and the small sample size, it is difficult to conduct these studies on mediastinal tumors.

Dynamic contrast enhanced MRI (DCE-MRI) is a non-invasive functional imaging method, which evaluates tumor blood perfusion and microvascular permeability by monitoring dynamic changes of MRI contrast agents in target tissues^16^. At present, DCE-MRI semi-quantitative and quantitative analysis has been widely used in the diagnosis, differential diagnosis, prognosis and efficacy evaluation of various solid tumors^17–20^. Shen et al.^20^ previously analyzed 29 patients with thymic carcinoma and thymic lymphoma, and found that DCE-MRI quantitative parameters had significant differences in thymic carcinoma and thymic lymphoma.

Texture analysis can quantitatively analyze the gray distribution characteristics, pixel relations and spatial features of images, and the extracted features can quantify the heterogeneity of tumors^21^. The assessment of tumor heterogeneity by texture analysis imaging has become a non-invasive tool for diagnosis, prognosis and treatment response in clinical Settings, such as breast cancer, prostate cancer, glioma or other solid tumors^22–24^. It is well known that thymic carcinoma is mainly squamous cell carcinoma with clear-cut atypia^25^. Lymphoma, on the other hand, is described in several previous reports to have homogeneous signal intensity due to the intratumoral characteristics of high cellular density, a small amount of stromal tissue and less micro-necrosis^26^. Previous studies have confirmed that thymic carcinoma is more heterogeneous than thymic lymphoma^10,20^. The value of DCE-MRI texture analysis has been confirmed in breast cancer, glioma, etc.^24,27^, but no relevant results have been found in the differentiation of thymic carcinoma and thymic lymphoma.

The purpose of this study was to investigate the usefulness of texture analysis of DCE-MRI for distinguish between thymic carcinoma and thymic lymphoma.

## Materials and methods

### Patients

This retrospective study was approved by the Institutional Review Board of Jiangsu Province People's Hospital and Nanjing Medical University First Affiliated Hospital, and the requirement to obtain informed patient consent was waived (Permit Number: 2021-SR-238). All methods were performed in accordance with the relevant guidelines and regulations.

In this retrospective study, we reviewed the medical records of patients with thymic carcinoma and thymic lymphoma in our hospital from April 2018 to March 2021. The patients who met the following criteria were enrolled: (1) the primary tumours were all confirmed by surgery or percutaneous puncture biopsy; (2) routine MRI and DCE-MRI parameters were complete; (3) no operation, puncture, radiotherapy or endocrine therapy was performed before MRI examination. We excluded 12 patients due to the following reasons: (1) inadequate MRI quality (n = 4); (2) treated before the examination (n = 8). Finally, we included a total of 68 pathologically diagnosed patients, including 32 patients with thymic carcinoma (22 males, 10 females, mean age 55.4 ± 13.1 years) and 37 patients with thymic lymphoma (18 males, 19 females, mean age 36.4 ± 14.9 years).

### Imaging protocol

All MRI examinations were performed using a 3 T MRI system (MAGNETOM Skyra, Siemens Healthcare, Erlangen, Germany) with a 16-channel torso coil. All patients underwent conventional MRI and DCE-MRI from the suprasternal notch to the diaphragm. Axial DCE-MRI used the StarVIBE sequence that enabled the patient to breathe freely. The conventional imaging protocols included an unenhanced axial T1-weighted imaging (140 ms repetition time (TR), 2.5 ms echo time (TE)) and coronal T2-weighted imaging (1200 ms TR, 93 ms TE). A bolus of gadolinium-diethylene triamine pentacetic acid (Magnevist; Bayer Schering Pharma AG, Berlin, Germany) was injected through the elbow vein via a power injector with a flow rate of 4.0 mL/s at the dose of 0.1 mmol/kg, followed by a 20 ml bolus of saline administered at the same injection rate. During the entire acquisition process, First, three non-enhanced datasets were acquired using T1W imaging starVIBE with flip angles of 5°, 10°, and 15°, respectively, to obtain the T1 map. Second, the dynamic sequence was acquired after T0 baseline acquisitions and thirty-one contrast-enhanced image sets were acquired. The StarVIBE DCE-MRI detailed imaging parameters were as follows: 3.19 ms TR/1.13 ms TE, 3 mm section thickness, 400 mm^2^ field of view (FOV), 160*224 matrix, 15° flip angle. The temporal resolution was 8.8 s, and the total acquisition time was 5 min 8 s.

### Imaging processing

DCE-MRI data were uploaded and processed with an in-house software (Omin-Kinetics; GE Healthcare, Shang Hai, PR China). For the selection of the arterial input function (AIF), a free-hand region of interest (ROI) was placed in the descending aorta on DCE-MRI images. The mean size of the ROIs ranged from 6–9 mm^2^. The AIF curve was approved by a senior chest radiologist to ensure its accuracy. The Extended Tofts Linear two-compartment model was used to calculate the pharmacokinetic parameters. Determine the location of the lesion by combining T2WI and DCE, adjust the image to the phase with the most obvious enhancement, draw the ROI on each cross section manually, and fuse the lesions in the software to generate the three dimensional ROI (3D-ROI). Measurement was carried out along the edge of the lesion tissue, ensuring that ROI was smaller than that of the lesion, reducing the effect of volume effect and making the lesion tissue in the region of interest more representative. The necrotic, cystic and bleeding areas should be avoided as far as possible. DCE-derived parametric maps, including the volume transport constant (K^trans^), plasma rate constant (K_ep_), and the extracellular space volume percentage (V_e_) were calculated based on the Tofts model automatically. The texture parameters were acquired using the same software (Omin-Kinetics; GE Healthcare, Shang Hai, PR China). Features utilized in our study include mean, median, 5th/95th percentile (P5/P95), skewness, kurtosis, diff-variance, diff-entropy, contrast and entropy.

Texture analysis of DCE-MRI images was performed by two experienced chest radiologists with 7 and 3 years of experience, both of them blinded to the clinical information and final histopathological results. The measurements of the two readers were used for the evaluation of the interobserver reproducibility.

### Statistical analysis

All statistical analyses were performed using the SPSS software package (version 26.0, Chicago, IL, USA) and MedCalc (version 20.0.4, Mariakierke, Belgium). The normality of data distributions was analyzed using the Kolmogorov–Smirnov test. All numeric data with normal distributions were reported as mean ± standard deviation. Otherwise, medians (25th–75th percentile) were reported. Independent sample t-test or Mann–Whitney U test was used to compare the differences in texture parameters between the two groups. Logistic regression was used to screen parameters and receiver operating characteristic (ROC) curve was used to evaluate the diagnostic value of each parameter in differentiating thymic carcinoma and thymic lymphoma. *P* < 0.05 were determined to be of statistical significance.

The inter-observer reproducibility of parameters measurement in this study were assessed using intraclass correlation coefficient (ICC) with 95% confidence intervals (CIs) and applying a two-way ICC with random rater assumption. The ICC was interpreted as follows: < 0.40, poor; 0.40–0.60, moderate; 0.61–0.80, good; > 0.81, excellent.

## Result

A significant difference was observed in patient age between the two groups (*p* < 0.001), while no differences on distribution of patient gender (*p* > 0.05) (Table 1). The pathological classification of the thymic carcinoma and lymphoma groups was shown in Table 1.

**Table 1:** Clinical characteristics of the patients.

Prameters	Thymic carcinoma (n = 32)	Thymic lymphoma (n = 37)	*P* value
Age	54.31 ± 12.25	36.95 ± 15.51	< 0.001
Gender (male/female)	22/10	18/19	0.092
Pathologic types (n)	SCC (29)	HL (16)	
	Adenocarcinoma (3)	DLBCL (13)	
		TLL (5)	
		MALT Lymphoma (2)	
		MCL (1)	

*SCC* squamous cell carcinoma, *HL* Hodgkin lymphoma, *DLBCL* diffuse large B-cell lymphoma, *TLL* T-cell lymphoblastic lymphoma, *MALT Lymphoma* Mucosa associated lymphoid tissue lymphoma, *MCL* Mantle cell lymphoma.

**Figure 1:**
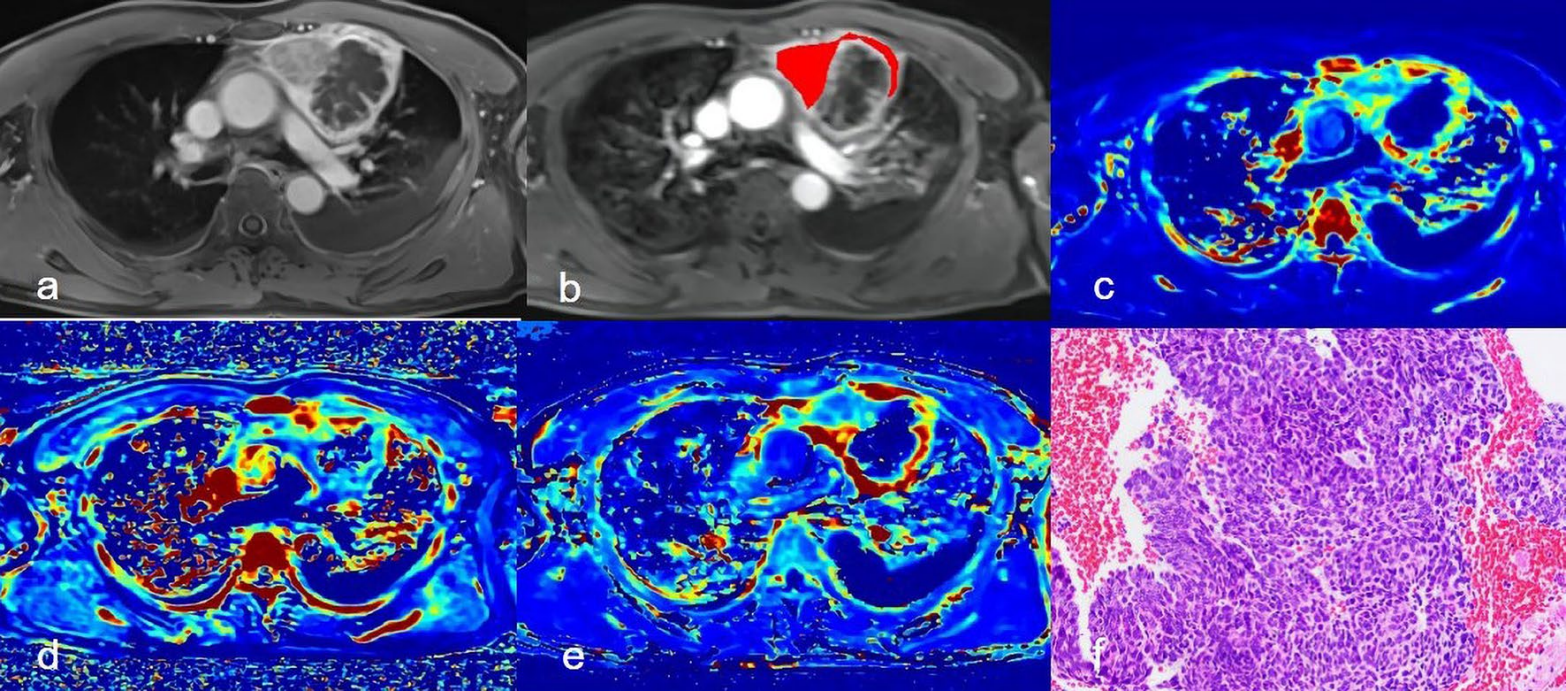
A 49-year-old man with thymic carcinoma (**a**) Dynamic contrast-enhanced scan showed anterior mediastinal mass, with cystic necrotic areas inside after enhancement; (**b**) Delineated the lesion; (**c**-**e**) Correspond to the generated Ktrans Map, Kep Map, and Ve Map; (**f**) Pathological sections (HE staining, 200 times magnification).

**Figure 2:**
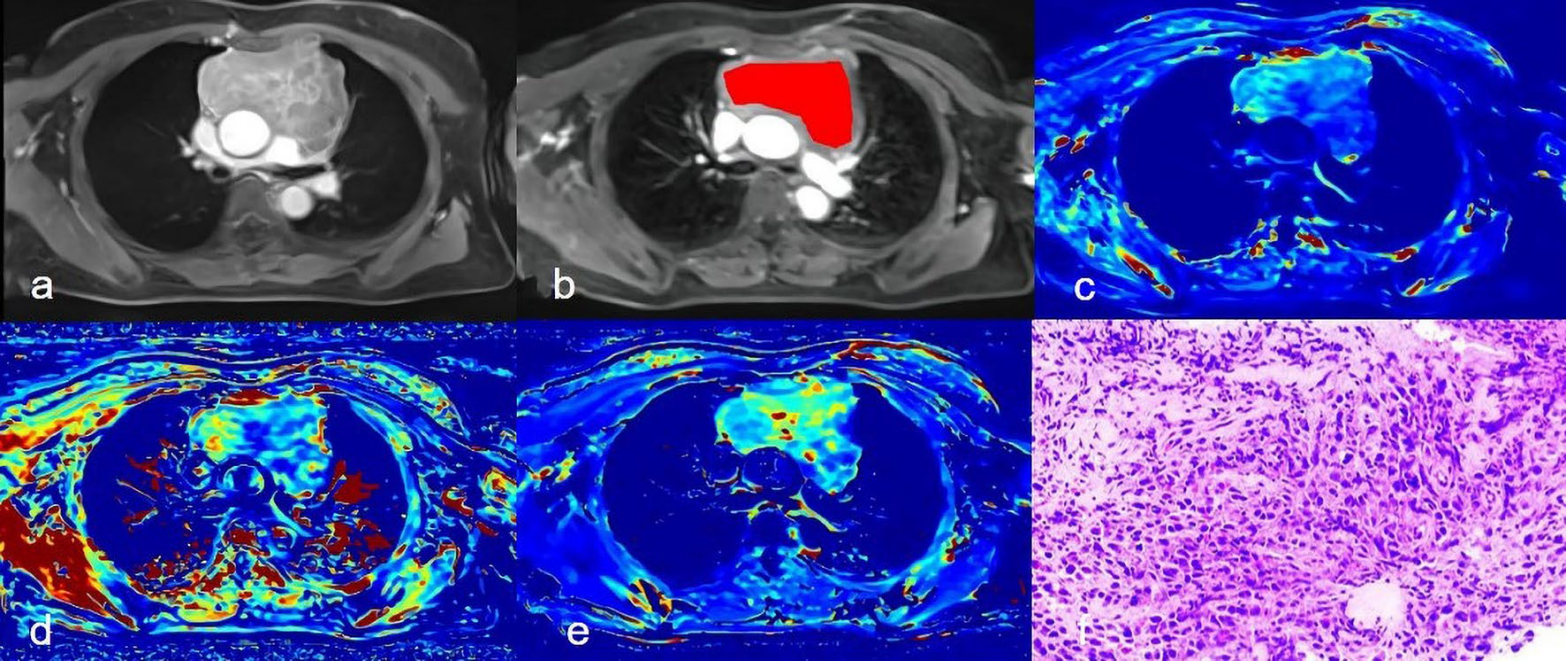
A 52-year-old female with Hodgkin's lymphoma (**a**) Axial position of dynamic enhanced scan showed anterior mediastinal mass, and uneven enhancement of the lesion after enhancement; (**b**) Delineated the lesion; (**c**-**e**) Correspond to the generated Ktrans Map, Kep Map, and Ve Map; (**f**) Pathological sections (HE staining, 200 times magnification).

Detailed comparisons of DCE-MRI texture parameters of both groups were summarized in Table 2. Their abilities to distinguish between thymic carcinoma and thymic lymphoma are shown in Table 3. Representative cases are shown in Figs. 1 and 2.

**Table 2:** DCE-MRI texture-derived parameters of the thymic carcinoma and lymphoma groups.

Characteristics	Thymic carcinoma (n = 32)	Thymic lymphoma (n = 37)	*P* value
***K***^***trans***^			
Mean	0.279 ± 0.114	0.220 ± 0.096	0.026*
Median	0.268 ± 0.104	0.208 ± 0.096	0.017*
P5	0.090 (0.054–0.134)	0.064 (0.044–0.135)	0.512
P95	0.479 ± 0.107	0.404 ± 0.131	0.014*
Skewness	1.152 (0.799–2.036)	1.305 (0.863–2.584)	0.665
Kurtosis	4.447 (2.125–8.162)	4.42 (1.832–19.541)	0.572
Diff-variance	0.000257 (0.000179–0.000428)	0.000274 (0.000176–0.000520)	0.880
Contrast	0.000455 (0.000343–0.000712)	0.000302 (0.000212–0.000625)	0.033*
Diff-entropy	0.342 ± 0.072	0.309 ± 0.078	0.086
Entropy	6.424 ± 0.693	5.960 ± 0.819	0.016*
***K***_***ep***_			
Mean	0.752 ± 0.229	0.579 ± 0.259	0.004*
Median	0.701 ± 0.199	0.539 ± 0.251	0.005*
P5	0.222 (0.157–0.312)	0.177 (0.103–0.306)	0.351
P95	1.011 (0.852–1.245)	0.898 (0.679–1.264)	0.250
Skewness	0.855 (0.350¬-1.603)	1.040 (0.520–1.907)	0.263
Kurtosis	1.752 (0.616–4.147)	1.883 (0.689–10.556)	0.186
Diff-variance	0.000821 (0.000608–0.001318)	0.000666 (0.000567–0.001367)	0.324
Diff-entropy	0.000405 (0.000333–0.000630)	0.000302 (0.000212–0.000625)	0.092
Contrast	0.423 ± 0.036	0.393 ± 0.059	0.015*
Entropy	6.688 ± 0.453	6.086 ± 0.751	< 0.001*
***V***_***e***_			
Mean	0.494 ± 0.180	0.376 ± 0.200	0.014*
Median	0.437 (0.338–0.624)	0.322 (0.188–0.494)	0.019*
P5	0.239 ± 0.123	0.197 ± 0.136	0.201
P95	0.753 ± 0.146	0.587 ± 0.202	< 0.001*
Skewness	1.034 (0.381–1.516)	0.567 (-0.251–1.327)	0.229
Kurtosis	2.539 (0.673–6.896)	1.641 (0.188–5.015)	0.220
Diff-variance	0.001569 (0.000846–0.004472)	0.002621 (0.001201–0.007933)	0.253
Diff-entropy	0.000527 (0.000375–0.001259)	0.000909 (0.000425–0.00240)	0.234
Contrast	0.426 ± 0.061	0.382 ± 0.074	0.010*
Entropy	6.946 ± 0.634	6.455 ± 0.708	0.004*

*Significant differences.

**Table 3:** ROC analyses of DCE-MRI quantitative parameters.

Parameters	AUC (95%CI)	Cut-off	Sensitivity	Specificity
K^trans^	0.769 (0.651–0.862)	0.313	0.906	0.514
K_ep_	0.780 (0.664–0.871)	0.440	0.875	0.622
V_e_	0.807 (0.694–0.892)	0.531	0.750	0.784
K^trans^ + K_ep_ + V_e_	0.962 (0.886–0.993)	0.438	0.938	0.892

K^trans^ represents all K^trans^ -related features that showed significant differences in univariate analysis (i.e., mean, median, P95, contrast, and entropy). K_ep_ represents all K_ep_ -related features that showed significant differences in univariate analyses (i.e., mean, median, entropy, and diff-entropy). V_e_ represents all V_e_-related features that showed significant differences in univariate analyses (i.e., mean, median, P95, entropy, and diff-entropy).

The mean, median, P95, and entropy of the K^trans^-related parameters were significantly higher in the thymic carcinoma than in the lymphoma (*p* = 0.026, 0.017, 0.014, 0.016, respectively), while the contrast of the K^trans^-related parameter was significantly higher in lymphoma than in the the thymic carcinoma (*p* = 0.033). The mean, median, entropy, and diff-entropy of the K_ep_-related parameters were significantly higher in the thymic carcinoma than in the lymphoma (*p* = 0.004, 0.005, < 0.001, 0.015, respectively). With regard to the Ve-related parameters, mean, median, P95, entropy, and diff-entropy were significantly higher in the thymic carcinoma than in the lymphoma (*p* = 0.014, 0.019, < 0.001, 0.004, 0.010, respectively).

**Figure 3:**
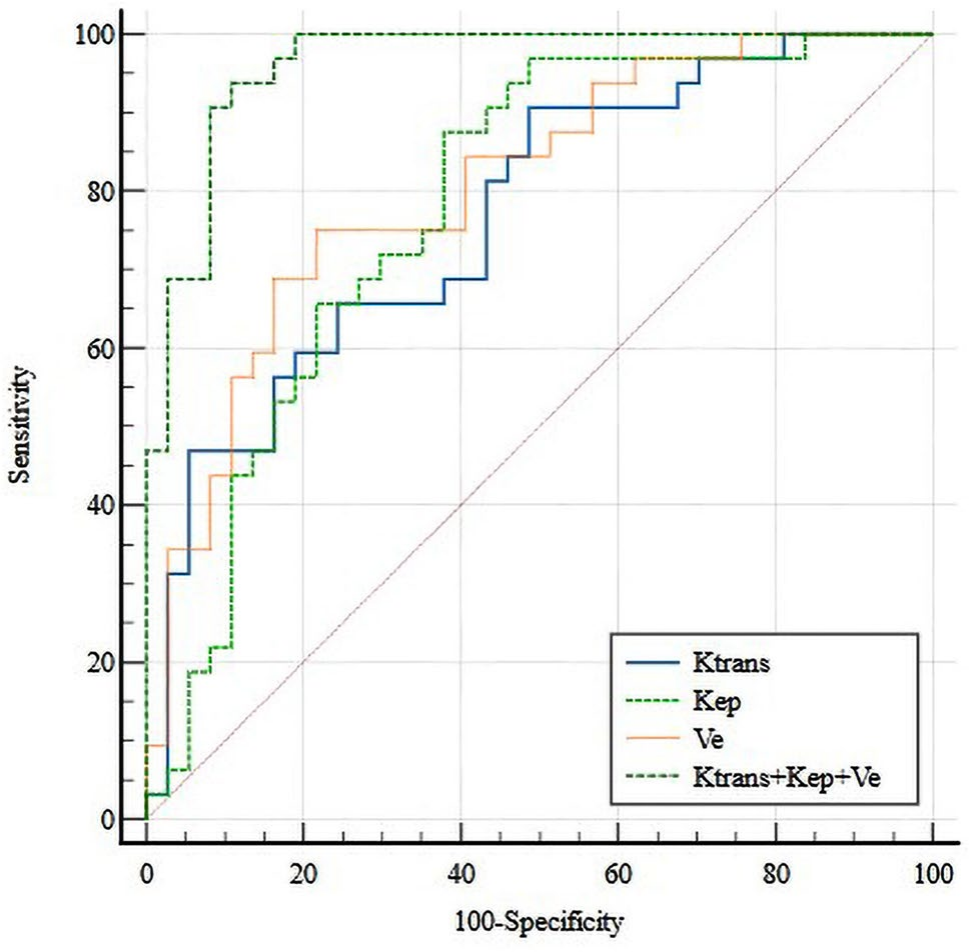
ROC curve of important texture-derived parameters in differentiating between thymic cancer and thymic lymphoma Groups.

The K^trans^-related significant features (including mean, median, P95, contrast and entropy) yielded an AUC of 0.769 (sensitivity 90.6%, specificity 51.4%) for the differentiation between thymic carcinoma and thymic lymphoma. We obtained an AUC of 0.780 (sensitivity 87.5%, specificity 62.2%) for the differentiation between the two groups with all these significant K_ep_-related features (including mean, median, entropy, and diff-entropy). Differentiation between thymic carcinoma and thymic lymphoma by using Ve-related significant features (including mean, median, P95, entropy, and diff-entropy) yielded an AUC of 0.807 (sensitivity 75.0%, specificity 78.4%). The combination of significant DCE-MRI textural features yielded an AUC of 0.962 (sensitivity 93.8%, specificity 89.2%) for the differentiation between thymic carcinoma and thymic lymphoma. Details are shown in Table 3 and Fig. 3.

**Figure 4:**
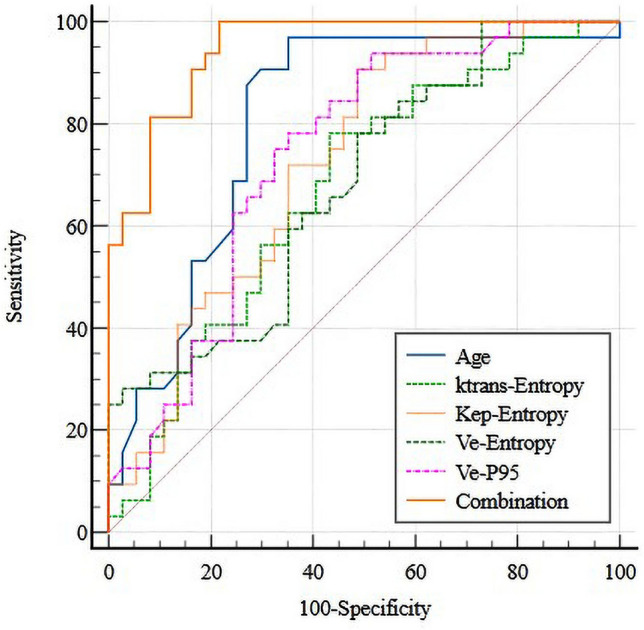
ROC curve of DCE-MRI quantitative parameters in differentiating between thymic cancer and thymic lymphoma Groups.

Five parameters were screened out after logical analysis, including age (AUC of 0.806, 96.9% sensitivity, 64.9% specificity), K^trans^-entropy (AUC of 0.671, 78.1% sensitivity, 56.8% specificity), K_ep_-entropy (AUC of 0.723, 90.6% sensitivity, 51.4% specificity), V_e_-entropy (AUC of 0.679, 78.1% sensitivity, 51.4% specificity), and V_e_-P95 (AUC of 0.738, 78.1% sensitivity, 64.9% specificity). The combination of those five parameters exhibited the better diagnostic performance in the determination of thymic carcinoma from lymphoma (AUC of 0.943, 93.7% sensitivity, 81.1% specificity). ROC curve analysis results were shown in Table 4. The ROC curves regarding texture parameters to differentiate thymic carcinoma from thymic lymphoma are shown in Fig. 4.

**Table 4:** ROC analyses of texture-derived parameters in differentiating between thymic carcinoma and lymphoma groups.

Parameters	AUC (95%CI)	Cut-off	Sensitivity	Specificity
K^trans^- Entropy	0.671 (0.547–0.779)	5.971	0.781	0.568
K_ep_-Entropy	0.723 (0.602–0.824)	6.155	0.906	0.514
V_e_-Entropy	0.679 (0.555–0.786)	6.507	0.781	0.514
V_e_-P95	0.738 (0.618–0.836)	0.642	0.781	0.649
Age	0.806 (0.693–0.891)	37	0.969	0.649
Combination*	0.948 (0.867–0.987)	0.344	0.937	0.811

*Combination represents the combination of age, K^trans^-Entropy, K_ep_-Entropy, V_e_-Entropy, and V_e_.

The two groups of data achieved good repeatability within and between observers (ICCs, ranges from 0.769 to 0.907).

## Discussion

In clinical practice, conventional MRI and DWI are not always accurate in the identification of mediastinal tumors. Conventional MRI cannot provide reliable and comprehensive information on tumor physiology such as microvascularity, angiogenesis, or metabolism, all of which are also important in the identification of tumor types^28^. Previous studies have confirmed that tumor blood volume and permeability obtained by DCE-MRI imaging technology have been found to be related to tumor type and degree of malignancy^29^. Tumors are heterogeneous at both genetic and histopathological levels, and heterogeneity exists in the number of cells, angiogenesis, extracellular matrix and necrotic area in different tumors^16^. It is important to assess tumor heterogeneity because tumors with high intra-tumor heterogeneity have poor prognosis, which may be secondary to inherent invasive biology or therapeutic resistance^30^. The value of DCE-MRI texture analysis in evaluating tumor heterogeneity has been widely demonstrated^24,27,31^. Shen et al.^20^ previously confirmed the value of DCE-MRI in distinguishing thymic carcinoma and lymphoma, but it could not adequately explain the heterogeneity of thymic carcinoma and thymic lymphoma.

In our study, we found that age and most of the DCE-MRI-derived texture parameters had significant differences between thymic carcinoma and thymic lymphoma. Age was significantly different between thymic carcinoma and thymic lymphoma (*P* < 0.05). It was reported that thymic lymphoma was more common in young people and thymus carcinoma was more common in middle-aged and elderly people^1,2,7^, which was consistent with our study. The texture parameters with significant differences in K^trans^, K_ep_ and V_e_ were combined and the diagnostic efficiency was calculated. It was found that V_e_ had higher diagnostic efficiency. Combine all of the above parameters, the differential performance was improved with an AUC of 0.962, confirming the important value of DCE-MRI texture parameters in differentiating thymic carcinoma and thymic lymphoma. After logistics regression screening, four texture parameters are obtained, namely, K^trans^-entropy, K_ep_-entropy, V_e_-entropy and V_e_-P95. Combined age with K^trans^ -entropy, K_ep_-entropy, V_e_-entropy and V_e_-P95, the identification efficiency was significantly improved with the AUC was 0.943.

V_e_ can indirectly reflect the density of tumor cells, which is inversely proportional to the density of tumor cells^16^. In general, tumors with a high degree of malignancy are histologically characterized by dense cell density, reduced extracellular space and dense lesion structure, resulting in a corresponding decrease in ADC value reflecting cell density^32^. Previous studies have shown that lymphoma is a cell-rich tumor composed of atypical lymphocytes of uniform size, while thymic carcinoma often presents uneven signal due to internal cystic degeneration, necrosis or hemorrhage^20,25,26^. Zhang et al.^10^ studied 15 cases of thymic carcinoma and 13 cases of thymic lymphoma and found that the ADC value of thymic lymphoma was significantly lower than that of thymic carcinoma, which may increase the V_e_ parameters in the thymic carcinoma, the mean, median, P95 and entropy of V_e_ in thymic carcinoma group were higher than those in thymic lymphoma group (P < 0.05) in this study. Entropy is one of the most commonly used and effective texture features, which refers to the disorder degree of pixel intensity relationship in ROI^33^. The higher the entropy, the higher the heterogeneity. Diff-entropy is a random measure of the gray difference between adjacent voxels. The relationship between heterogeneity and diff-entropy is not clear. In previous studies, we found that the value of diff-entropy was higher in the lesions with a higher degree of malignancy^31^, which was consistent with our findings.

Both K^trans^ and K_ep_ are positively correlated with vascular permeability and angiogenesis in tumor tissues. The vascular permeability of thymic carcinoma group is higher than that of thymic lymphoma group due to the abundance and immaturity of immature blood vessels and the incomplete structure of endothelial cells in neovascularization^20^. Shen et al. reported thymic carcinoma had significantly lower Kep than thymic lymphoma^20^. The differences of the former results may be clarified by the different cohorts of the different thymic and lymphoma patients. Some low potential malignancy lymphoma patients like mucosa associated lymphoid tissue lymphoma was included in our study while it was not enrolled in the former study^20^. A further study to compare the different subtypes of lymphoma may be more meaningful in the future. Therefore, the mean, median, entropy, and diff-entropy of K_ep_ in the thymic carcinoma group were higher than those in the thymic lymphoma group in this study and the mean, median, P95, entropy, and diff-entropy of K^trans^ related parameters are significantly higher in thymic carcinoma than in thymic lymphoma.

The present study had some limitations. First, there was a relatively small sample size for this study. A further study with more patients is needed to reinforce the statistical persuasiveness. Second, the ROI of the lesions in this study was manually delineated, and the microscopic cystic changes, necrosis and bleeding areas inside the tumor were inevitable, which may have certain influence on the results. Third, the imaging time was long and the post-processing analysis was complex, which make the proposed method infeasible for clinical application. Finally, texture features used in our study are relatively simple. Further studies including more comprehensive texture features would be a meaningful topic.

In conclusion, whole-lesion histogram and texture analyses of parameters derived from DCE-MRI may be of value in differentiating thymic carcinoma from thymic lymphoma. Using texture analyses, DCE-derived features can be assessed as potential biomarkers for differentiating between thymic carcinoma and thymic lymphoma.

## Data Availability

The datasets generated during and/or analysed during the current study are available from the corresponding author on reasonable request.
